# A Qualitative Study of Stress and Coping to Inform the LEADS Health Promotion Trial for African American Adolescents with Overweight and Obesity

**DOI:** 10.3390/nu13072247

**Published:** 2021-06-29

**Authors:** Mary Quattlebaum, Colby Kipp, Dawn K. Wilson, Allison Sweeney, Haylee Loncar, Asia Brown, Sydney Levine, Nicole Zarrett

**Affiliations:** 1 Department of Psychology, University of South Carolina, Columbia, SC 29208, USA; mjq@email.sc.edu (M.Q.); ckipp@email.sc.edu (C.K.); gauseh@email.sc.edu (H.L.); asia@email.sc.edu (A.B.); levinesm@email.sc.edu (S.L.); zarrettn@mailbox.sc.edu (N.Z.); 2 College of Nursing, University of South Carolina, Columbia, SC 29208, USA; sweeneam@mailbox.sc.edu

**Keywords:** stress, health behaviors, coping, African American adolescents

## Abstract

The purpose of this study was to conduct in-depth individual interviews with 30 African American adolescents with overweight and obesity and their families (caregiver/adolescent dyads) to gain a better understanding of how to integrate stress and coping essential elements into an existing family-based health promotion program for weight loss. Interview data from 30 African American adolescents with overweight and obesity (*M*age = 15.30 ± 2.18; *M*BMI%-ile = 96.7 ± 3.90) were transcribed and coded for themes using inductive and deductive approaches by two independent coders. Inter-rater reliability was acceptable (*r* = 0.70–0.80) and discrepancies were resolved to 100% agreement. The themes were guided by the Relapse Prevention Model, which focuses on assessing barriers of overall coping capacity in high stress situations that may undermine health behavior change (physical activity, diet, weight loss). Prominent themes included feeling stressed primarily in response to relationship conflicts within the family and among peers, school responsibilities, and negative emotions (anxiety, depression, anger). A mix of themes emerged related to coping strategies ranging from cognitive reframing and distraction to avoidant coping. Recommendations for future programs include addressing sources of stress and providing supportive resources, as well as embracing broader systems such as neighborhoods and communities. Implications for future intervention studies are discussed.

## 1. Introduction

African American adolescents show disproportionately higher rates of overweight and obesity (40%) than their White counterparts (31%) [1]. Adolescent obesity has primarily been attributed to physical inactivity, sedentary behaviors, and increased consumption of energy-dense, highly palatable foods [2]. However, recent studies have investigated the relationship between high levels of adolescent stress and obesity-promoting behaviors, such as decreased physical activity, addictive eating, and other dieting behaviors [3,4,5]. Previous research on adolescent stress and health behaviors has revealed a variety of determinants related to stress and unhealthy behaviors, such as parent–adolescent conflict and daily stress related to school and racial discrimination [6,7,8,9,10]. Understanding which stress-related risk factors are most impactful for African American adolescents with overweight and obesity is critical for the continued effort to develop and implement efficacious prevention and treatment programs. 

Current conceptual frameworks for stress and coping based on Lazarus and Folkman [11] have defined stress as an individual’s experience/perception of a stressor that either matches or exceeds an individual’s ability to manage that situation. Chronic stress, in particular, has been shown to lead to negative biological, behavioral, and psychological outcomes. Given that African American families have a high level of chronic stressors due to various social and cultural factors, further research is needed to better understand stress and coping processes within the context of these families [12,13]. Prior research with African American families suggests that salient chronic stressors include family and interpersonal conflict, financial difficulties, racial discrimination or racism, community violence, and neighborhood disorder [13,14]. A key element of stress is the individual’s perspective and evaluation of potential harm [11,15]. When a situation/stressor is beyond the perceived coping level, the individual experiences negative emotions that could lead to unhealthy behavior, such as poor diet quality, physical inactivity, and prolonged screen time [11,16]. This may be particularly salient among African American families, given that they may have fewer resources to aid in effective coping strategies [17]. Thus, stress and coping processes may be important in understanding how to develop more effective programs to increase participation in and acceptability of health promotion programs in African American families. 

Few previous studies have examined the impact of stress-related factors on health behaviors, such as eating and physical activity specific to African American adolescents. A systematic review of all youth, including ethnic minorities, showed that 13 studies found a positive association between stress and unhealthy eating behaviors in adolescents, such as increased sugar intake [18]. Furthermore, previous studies have shown that African American adolescents, in particular, utilize avoidance strategies to cope with stress and have a lack of resources for coping with stress-related issues, which has been associated with unhealthy eating behaviors, such as overeating [19]. Importantly, race-related stressors have been linked to problematic eating behaviors among African American young adults [9,10]; however, minimal studies have assessed this relationship in younger minority samples. 

Several studies have also examined associations between chronic stress and physical activity in adolescents. For example, in a 5-year longitudinal study [20], investigators found that extrinsic life stressors (financial strain, lack of transportation) and personal life stressors (relationship conflicts, bereavement) were negatively associated with physical activity outcomes in youth. In a study by McGlumphy et al. [21], perceived stress was also significantly associated with lower levels of physical activity in African American boys, in particular. Specific coping strategies most frequently endorsed by adolescents included trying to stay calm, being alone, watching TV/listening to music, sleeping, and playing a game. Taken together, these studies demonstrate that the associations between stress and physical activity and diet are critical in understanding how stress may impact these health behaviors in African American adolescents with overweight and obesity.

While previous studies have demonstrated that African Americans experience higher levels of chronic stress and worse health outcomes, there has been a lack of understanding of how best to integrate stress management interventions into health promotion programs for these high-risk populations [22]. Stress management techniques and strategies may be useful for families in health promotion programs as stress presents as a barrier to engagement in healthy behaviors, such as diet and exercise, which in turn can impact overall physical and psychological well-being [23,24]. There has been some evidence showing that incorporating stress management techniques into health promotion interventions results in improvements for health-related outcomes and overall quality of life, however, few of these studies have included African American adolescents [25,26,27]. Furthermore, much of the past research has focused primarily on adults and non-African American populations. Improved understanding of stress and coping through the lens of African American families is needed, as these families experience unique chronic stressors, namely race-related stressors, and may benefit from culturally tailored coping strategies for these stressors [28]. Future research should focus on the pertinent and unique stress-related factors among African American adolescents to inform integrated stress management and health promotion interventions. Additional research is also needed to elucidate the role of resilience in relation to chronic stress and health outcomes among African American families, as this has been highlighted as a key protective factor among minority populations exposed to elevated risk [29]. This strengths-based framework may further elevate programs that integrate stress management and health promotion and include African American families.

The purpose of the current study was to obtain qualitative data to understand the lived experiences of African American adolescents with overweight and obesity, gain a richer understanding that expands beyond past quantitative results, and in this case use these insights to inform a health promotion intervention (Linking Exercise for Advancing Daily Stress Management (LEADS)). The quantitative studies reviewed above indicate that stress has a major impact on health-related behaviors, but there is still limited understanding of how African American adolescents with overweight and obesity view and approach stress and coping. Qualitative data may better highlight African American families’ unique experiences with stress and coping compared to prior quantitative studies. The current study relied on Bronfenbrenner’s ecological systems framework to guide interview questions, which allowed participants to disclose their lived experiences with stress and coping [30].

Integrating the perspectives/feedback from African American adolescents will ultimately help to make the LEADS intervention more likely to be feasible, accepted, and impactful. Specifically, this study used in-depth interviews to assess African American adolescents’ stressors, coping strategies, and the impact of these stressors on health-related behaviors. Individual interviews were conducted with adolescent participants with overweight and obesity following their completion of the Families Improving Together (FIT) for Weight Loss randomized controlled trial [31,32,33]. This qualitative study assessed adolescents’ (a) perception of stress, (b) sources of stress, (c) coping strategies, (d) perception of health behaviors related to stress, (e) social support (e.g., source and type of support), (f) community insights and (g) recommendations for future programs. Findings from the current study were designed to inform the development of the LEADS intervention, a health promotion and stress management intervention efficacy trial.

## 2. Materials and Methods

This study was conducted post-intervention of the FIT trial to obtain follow-up qualitative data on families’ perception of stress and coping, health behaviors, and recommendations for future health promotion programs. For the FIT trial, families participated in a 2-week orientation phase, and were then randomized to the initial phase of the trial that tested the efficacy of a face-to-face 8-week motivational plus family weight loss (M + FWL) program compared to a comprehensive health education (CHE) program. Then, families were re-randomized for the second phase of the trial that tested the efficacy of an online 8-week M-FWL program to an online CHE (See [31] for full details). The trial aimed to reduce BMI z-score (z-BMI) and improve diet and physical activity in African American adolescents (aged 11–16 years old) with overweight and obesity. The FIT intervention did not directly target stress or stress reduction behaviors as part of the intervention curriculum. Starting at 1-year post-intervention, FIT families were invited to participate in this follow-up study (*N* = 30), in which face-to-face individual interviews were conducted separately with parent-adolescent dyads. The FIT trial was conducted from 2013 to 2018 and consisted of 16 cohorts. The current study sample included participants in FIT cohorts 7 to 16, which were completed between 2015–2018. Interviews were conducted between November 2018 and February 2020, thus, participants may have completed their interviews for the current study up to 5 years after completing the FIT trial. Of the 30 participants in the current study, 20% (*n* = 6) completed the M + FWL program both face-to-face and online, 30% (*n* = 9) completed the M + FWL face-to-face and the CHE online, 30% (*n* = 9) completed the CHE face-to-face and the M + FWL online, and 20% (*n* = 6) completed the CHE both face-to-face and online. Thus, the representation of families from each condition is approximately equal. This study was approved by the University of South Carolina Institutional Review Board. Participants completed informed consent and assent forms prior to participating in the in-depth interviews. Participants’ families received $40 in exchange for their participation in the interviews.

### 2.1. Participants

Participants (*N* = 30) were African American adolescents that were previously enrolled in the FIT trial [31,32,33,34,35]. Participants from the more recent cohorts of the FIT trial conducted in 2015–2018 (~80 families) were contacted by phone and/or email offering them an additional research opportunity. Previous qualitative studies have had a similar number of participants [36]. Participants were eligible if they met the following criteria at their FIT baseline appointment: (1) African American adolescent between 11–16 years of age, (2) having overweight or obesity, as defined by having a BMI ≥85th percentile for their age and sex, (3) and having a caregiver willing to participate with the adolescent. At the time of their follow-up individual interviews, participants (50% female) ranged from 13–21 years of age (*M* = 15.30, *SD* = 2.18). At their FIT baseline appointment, participants on average had a BMI that placed them in the obese range (*M_z-BMI_* = 0.78, *SD* = 0.50; *M_BMI%-ile_* = 96.7, *SD* = 3.90), however, the sample included adolescents with overweight and obesity. Among adolescents’ caregivers, 20% were married or in a committed relationship and 83.3% were employed part or full-time. Additionally, 60% of adolescents’ caregivers reported yearly income below $40,000.

### 2.2. Procedures

Three trained graduate research assistants conducted interviews in person with the participants. Participants were also asked to complete a brief demographic survey before beginning the interview. The interviewers followed a standard protocol including introducing the purpose of the interviews, encouraging confidentiality of responses, defining key terms, and asking the questions and probes in an open-ended manner (See Table 1). The first few questions asked about participants’ perceptions of the meaning of stress, their sources of stress, consequences of stress, and how those stressors may affect their health behaviors. Next, participants were asked about how they coped with stress and from whom they received support. Additionally, participants were asked how they might change their community to support their families and what recommendations about stress management they might have to improve health promotion programs. The interview questions were designed with Bronfenbrenner’s ecological systems framework using a top-down approach to allow for participants to present topics related to stress, coping, and health that were salient to them and their lived experiences across various systems [30]. The ecological systems model is rooted in the understanding that an individual’s behavior is impacted by various levels and systems that extend beyond the individual, including interpersonal, community, environmental, and policy influences [36,37]. This model has informed the development of multilevel programs that target several relevant systems that influence health behaviors [38]. Thus, this approach was integrated into the current study to capture the various systems that influence stress, coping, and health in our sample of African American adolescents. Sessions lasted between 45–60 min. All sessions were audio-recorded and transcribed by an independent transcription company. 

Of note, the three interviewers identified as Caucasian, and thus interviewer-participant racial/ethnic discordance was present in the current study. In line with prior research on racial/ethnic concordance among patients and mental health clinicians or primary care providers [39,40], this discordance in the present study may have influenced participants’ comfortability or affect in their discussions of stress, coping, and health behaviors. However, interviews for the present study were conducted prior to several high-profile cases of racial injustice highlighted in the media, and thus these stressors may not have been as salient, although racial discrimination and racism have been well-established as chronic, everyday stressors [13]. Interviews were also conducted prior to the onset of the COVID-19 pandemic, which may have exacerbated several stressors within minority populations [41]. 

### 2.3. Analysis

Interview transcripts were reviewed using a combination of deductive and inductive approaches. A preliminary set of themes were compiled using existing models of stress and coping [11]. Themes were also informed by previous research on sources of stress in African American families [42,43] and theories regarding coping and resiliency [44]. Additionally, in order to assess stress and coping across multiple systematic levels, themes were informed by Bronfenbrenner’s ecological systems theory [30], including evaluating individual, interpersonal, and community-level factors. The ecological systems framework was utilized to allow for participants to disclose topics related to stress and coping that were important to them and to capture stress and coping through the lens of the families [30]. Experiences of stress and coping related to race were not explicitly probed by the interviewers in an effort to be respectful and sensitive to topics that families were comfortable discussing. Next, using an inductive approach, the transcripts were reviewed to look for other themes not captured in the preliminary a priori list of themes [45]. A codebook was then developed from this list of themes. In order to reach consensus on codes assigned to the adolescent transcripts, a coding team of two independent coders (2 graduate students) met weekly to discuss differences in code assignment and to assess the relevance of certain themes. Initially, two transcripts were used for training purposes to establish preliminary reliability between coders. After establishing initial reliability, independent coders coded all 30 adolescent transcripts. 

Interrater reliability between coders indicated an acceptable level of agreement (*r* = 0.70–0.80). Additionally, disagreements between coders were resolved through discussion conducted over multiple conversations until 100% consensus was reached. A third coder was utilized for any persistent disagreements, but these occurrences were minimal. The qualitative software Dedoose (V. 08) was used to perform a thematic content analysis [46]. The frequency of themes and sources were determined. Frequency of themes was defined as the total number of instances the theme was coded across all participants throughout the duration of the interview. A cluster of sentences fitting a certain theme were coded as a single frequency, rather than individual sentences within the cluster accounting for multiple frequencies. Frequency of sources was defined as the number of participants that endorsed each theme. Themes that were endorsed by half or more of participants were considered prominent themes; thus, these themes were described in-depth throughout the results section. Differences in themes based on age (<18 years old versus 18+ years old [*n* = 5]) and sex were assessed. There were no prominent differences in themes based on age, so the data were combined for all age groups. A priori themes that were not endorsed by any adolescents or were endorsed by only 1 adolescent were not reported in the results. 

## 3. Results

Table 2 provides the demographic information for the current study sample compared to the larger FIT trial sample. Overall, the comparison of demographics of this subsample and the full FIT sample are relatively equivalent in sample characteristics. However, this study sample included a significantly lower prevalence of parental marital status (16.7% subsample; 34.4% FIT; *p* = 0.05) compared to the full FIT sample. Table 3 provides a summary of the themes organized by frequency and the number of sources that endorsed the theme. The following categories were utilized to summarize the qualitative results: perceptions of stress, sources of stress, consequences of stress, perception of health behaviors related to stress, additional behaviors related to stress, coping strategies, social support (e.g., source and type of support), community insights, and recommendations to address stress in future health promotion programs for families. 

### 3.1. Perceptions of Stress

Adolescents were asked to define the meaning of stress, resulting in endorsement of several emotional consequences of stress, including frustration, anger, and worry. Nearly half of participants commented that their perception of stress was related to worry/anxiety, with one participant noting that stress can “make you overthink, and like you can’t really, you don’t know what to do and sometime[s] you might just [go] over the edge or you just stuck.” Another prominent theme described by participants included negative emotions related to stress (e.g., “There’s a little bit of frustration or anger that people have and could affect their mental health;” “Cause like, I been through a whole lot in my life and every time anyone brings it up I always cry or get mad, and I always have to have a stress ball just to calm myself down.”). Some participants described stress as an obstacle or poor circumstances and others noted that stress contributed to feelings of tension (e.g., pressure from self or others, stressors building up over time) or helplessness (e.g., wanting to give up, having difficulty understanding or accomplishing a task). 

### 3.2. Sources of Stress

Several sources of stress were identified by adolescent participants, with the most prominent themes related to school, extracurricular responsibilities, and family and friends. Over 85% of adolescents described school as a salient stressor, including managing several deadlines and understanding difficult content, getting good grades or progressing to the next grade, receiving minimal support or experiencing pressure from teachers, balancing school with other responsibilities, paying college tuition, and preparing for the future. One adolescent stated, “Like grades, making sure you have your work in and, like if you have, like a lotta projects at the same time and a lotta work at the same time, that can get, like really stressful.” Adolescents also commented on responsibilities outside of school as a source of stress, including demands from sports and other extracurricular activities (e.g., concerns about performance, time requirements, and negative feedback from coaches). 

More than half of adolescents also noted stress related to social factors, including peer or romantic relationships and interpersonal or family conflict. Adolescents described stressors with peer or romantic relationships, including peer pressure, disagreements, or bullying/teasing (e.g., “Well maybe, like drama within a friend group, maybe I like to hang out with somebody and someone else doesn’t wanna hang out with them. But I don’t wanna, like have the best of both worlds, so you know, that can be stressful inside of my hang out session”). Additionally, adolescents endorsed stress related to interpersonal or family conflict, including feeling pressure or disappointment from parents, strict parenting, negative family communication, helping with childcare responsibilities, disagreements with siblings/extended family members, or parent–child conflict related to chores, behavior, or schoolwork (e.g., “And she [participant’s caregiver] be like what’s wrong with you, and I don’t be wanting to tell people what’s wrong with me cause I feel like she gonna [be] picking about the situation, the whole family gonna know and I don’t want everybody knowing my business, I don’t like that. Like, what me and you talk about should be just me and you, she wanna tell the whole family or she just wanna bring me down, make me worse so I just don’t even say nothing to nobody, just let it build in me and cry it out.”). Additional themes related to family and homelife stressors were described, such as family health concerns, general family worry, and poor conditions within the home or neighborhood environment (e.g., crime, drugs, kidnapping/trafficking, limited space in the home, and keeping the home clean). 

### 3.3. Consequences of Stress

Adolescents commented on various physical and emotional consequences of stress. Specifically, more than half of adolescents described physical consequences of stress (e.g., headache, stomachache, body tension or pain, sluggish or tired, nauseous, shaky, sweaty, hyperventilation, increased blood pressure, hair loss, etc.). Notably, 90% of adolescents reported emotional consequences of stress on health behaviors, including “I guess to put out an example, you know, you go through a lotta these things such as, you know, stress eating and all these depressing or sad thoughts or maybe even rude or disrespectful thoughts to other people around your area, I guess that would be something that would affect your health and overall everything. And if you don’t stop it it’ll definitely be in there for the rest of your life, you never change as a person.” 

### 3.4. Health Behaviors

Adolescents described the impact of stress on their health behaviors (e.g., eating behaviors, physical activity, sleep). Many adolescents noted the influence of stress on various health behaviors, including increased sedentary behavior and poor eating behaviors (e.g., consuming low dietary quality foods, overeating, undereating, stress eating or emotional eating). Interestingly, some adolescents noted that stress contributed to lower physical activity, whereas others commented on increased physical activity. Similarly, several adolescents endorsed sleeping excessively while others indicated difficulty getting enough sleep. Notably, some participants commented on their family’s response to stress with poor health behaviors, such as excessive sleep, overeating, and physical isolation (e.g., “Like my dad, he loves potato salad so he’ll eat, like a lotta potato salad, or he’ll do something really unhealthy when he’s like distressed I think.”).

### 3.5. Additional Behaviors

Several participants reported engaging in other behaviors in response to stress that were not related to health behaviors, with prominent themes related to avoidance behaviors and increased media usage. Specifically, some adolescents reported more avoidant responses to stress, including avoiding talking or seeing others, hiding one’s emotions, distancing themselves from their negative thoughts or emotions, and trying to think of something other than their stress. Some participants commented on consuming more media and screen time in response to stress, including watching T.V. or videos or using their phone. 

### 3.6. Coping Strategies

Adolescents endorsed several coping strategies for dealing with stress, with more than half of the participants commenting on engaging in various behavioral, cognitive, and social approaches to coping. Notably, no adolescent participants endorsed coping strategies related to environmental support (e.g., neighborhood safety and cohesion, access to resources for exercise and healthy foods). Several adolescents noted behavioral coping strategies, such as physical activity, listening to music, taking time to rest, and spending time alone. Additional behavioral coping strategies included reading or writing and screen time (TV, phone, online games).

Regarding cognitive approaches to coping, more than half of adolescents engaged in passive cognitive coping strategies, including avoiding or distancing themselves from thoughts or negative emotions related to stress (e.g., “I normally keep it to myself. I don’t like sharing my feelings. Like I barely cry and if I do cry I don’t like crying in front of people. I think it shows my weakness and I don’t like doing that.”). Interestingly, while some adolescents’ comments on distraction from stress appeared to demonstrate more passive coping strategies (e.g., “And so I was stressed about my EOC [exam] cause they weren’t, like putting my grades in and I was like, oh my gosh, what if I don’t, like pass. So I start to, like hide stuff, like I start to hide, like my charger so my mind would get off of it and I would, like look for my charger all day, then I’ll go to sleep. Even though, like I know where it is, I just act like I don’t know where it is. It’s like I act like I don’t know stuff sorta. To, like get me away from the reality of the stress and everything, it’s whatever. It cools me down.”), others appeared to represent more active coping techniques (e.g., “Like I’ll start, I probably go outside and dribble the basketball, like run around, do something to take my mind off of it.”). 

A few adolescents described other cognitive coping strategies that appeared to be more adaptive approaches, including contemplation (e.g., “Like it help me think about how I could’ve changed the situation instead of getting upset about it.”), utilizing positive thinking (e.g., “So, like I’m super stressed I’ll just, like lay down, try to calm myself down, like think about it, try to put positive thoughts in my head, not negative ones.”), and modifying or reframing their thoughts about stress (e.g., “so I tried to, like you know, train myself up or something because I know that I’m stressed and I don’t need to be this stressed as a young person, I don’t need to be this stressed.”). Several adolescents also reported meditation and mindfulness, and others noted prayer as a cognitive coping strategy. 

Prominent themes related to social support as a coping strategy included adolescents’ spending quality time with and talking to others. Specifically, many adolescents commented on hanging out with friends and fewer adolescents commented on time spent with family members. Half of adolescent participants discussed talking with others (family, friends, etc.) as a strategy for coping, with one participant sharing, “Either I probably go try, like a good person, like my momma, I talk to her about some things but not really too much. And, like my dad he’ll come in when he feel the need to, and so I can talk to him, just get it off me.”

### 3.7. Sources of Social Support and Types of Social Support

When asked what sources of social support adolescents depended on when faced with stressors, adolescents described parents, friends, and extended family as prominent themes. Additionally, some adolescents commented on social support from their church and school. Of note, several adolescents reported limited social support generally or limited community or neighborhood support, suggesting a need for additional social resources within the family system and broader community microsystem to effectively manage stress. Specifically, adolescents reported managing stressors on their own for various reasons, including concerns that seeking support would cause conflict, add stress to others, be unhelpful, no support is available, or concerns of feeling uncomfortable disclosing negative emotions and stress to others. 

One participant commented, “That’s kinda like really, I don’t really talk to nobody about it because I didn’t, like, you know, like how some people I used to, like, you know, vent to or, you know, rely on or whatever. Not, you can’t really, like talk to everybody, I guess that’s what I can say, cause not everybody’s truly going to wanna be there for you. So I just personally deal with it on my own.”). Additionally, adolescents noted a lack of support within their community due to limited communication, undeveloped relationships, or discomfort disclosing stressors with community members, with one participant stating that, “They’re support but not when we’re stressed. Like as a community, like the connection is there but when it comes to like when we’re stressed, it’s not like hey, it’s okay, it’s okay to be stressed. It’s not like that. Cause when we’re stressed we really don’t tell people cause it’s like, at that point it’s a personal issue and nobody says, well today me and my mom we had a, like a boxing match because, like we’re both stressed. It’s not like that.”

Adolescents reported various types of social support as salient themes, including emotional support, informational support, and communication. The role of emotional support included spending time with others, verbal and nonverbal affection, encouragement, and trying to improve one’s mood (e.g., “She definitely, you know, talks to me about the more positive things in life and, you know, explains that there will be negative aspects in life as you get older. And she’s very much more of the kinder, you know, more a warm source to talk to cause obviously that’s my mother. I still do have some talks with other family members but it’s not as, you know, close as it is with my mother.”). The role of informational support included others providing adolescents with advice, problem-solving, and educational resources. The role of communication and listening as a source of support included others listening to adolescents’ concerns, providing kind words, and discussing topics unrelated to a stressor. A few participants described tangible support and spiritual support, including transportation, food, academic support, childcare, home supplies, or gifts, as well as sharing scriptures or praying for families.

### 3.8. Community Insights

When asked how adolescents might adjust their community to better support them and their family in managing stress, suggestions provided by adolescents primarily focused on financial and tangible support, as well as constructing a community center. More than half of adolescent participants commented on financial support, indicating that they might use the funds to donate to charities, local churches, family members, or provide scholarships to students (e.g., “I guess if I were to have money I’d put it in places where there wouldn’t have to be stressful situations anymore or—solving money problems is one thing. But sometimes stress can do stuff that money can’t solve exactly.”). Nearly half of adolescents discussed tangible support, such as providing school supplies, food, affordable housing, (re)building and cleaning up infrastructure in the community schools, parks, and neighborhoods, and providing childcare resources (e.g., “Being raised by, like a single mother for most of my life I would love to build, like affordable housing for single mothers to live in so they wouldn’t have to have the stress of keeping lights on and, you know, feeding their child and, you know, just making sure that they can take care of their child and they can live their life. And the child doesn’t know struggle, they just know peace.”). Building a community center was another important theme, with one adolescent stating, “…probably like a community center. So like, it could be, like games and stuff and have, like daycare for kids, you could have therapy sessions, stuff like that so that people can feel comfortable to just come there whenever they want and relax and stuff, get stress off their mind. Have, like an exercise room cause I know some people they exercise to cope with stress. Have a café, but not thing, like no fast food cause I know some people are stress eaters like me. You could have, like home cooked food.” Other themes regarding community support included promoting peer support, building neighborhood cohesion, mental health support, and group-based programs and events. 

### 3.9. Program Recommendations

Adolescents provided suggestions of important elements to address stress in a family-based health promotion program, resulting in primary themes related to promoting positive health behaviors (e.g., physical activity, healthy eating), building coping skills, and receiving support from a mental health care provider. Over half of participants expressed interest in integrating healthy eating into programs to help manage stress, including a need for affordable healthy food options, access to fruits and vegetables rather than junk food to eat while stressed, educational resources related to stress and healthy eating, recipes and meal planning, preventing weight gain, and utilizing autonomy-supportive (rather than adding pressure or limits) approaches to recommend healthy eating. One participant commented that, “So like let’s say she gets, like stressed or something and she wants to eat, all you have to eat is just, you know, just like fruits and vegetables. So at least if you’re eating you’re gonna be eating good stuff.” Several adolescents noted the importance of opportunities to engage in physical activity in future health promotion programs, with a participant stating that “So, like if some people say they eat or they exercise or they listen to music or something like that, like I could include, basically all the things that they use to cope with stress I could include that in there, like the exercise room, to have, like a mini gym or a track. You could have, like a park outside, like a playground or something. A dance studio, cause some people like to dance, to listen to music to cope with stress.” 

A number of adolescents commented on building coping skills in future health promotion programs aimed at addressing stress, including education related to stress and coping, family resources for coping, and adaptive strategies to manage stress. One adolescent commented, “…[the] most effective ways to bring your stress levels down. I would also say, like never, I would say some tips to have, never do things when you’re stressed, like driving, drinking, eating, like make sure somebody else portions your food out while you’re stressed cause, you know, some people street eat. Like, have a list, like things that you shouldn’t do and the things that might affect you while you’re stressed.” Others suggested having an on-site therapist or counselor incorporated into a health promotion program to help cope with stressors. Building family skills was also a notable theme reported by adolescents, including exercising as a family and activities to disclose and problem-solve stressors as a family.

## 4. Discussion

The current study used qualitative analyses to assess perceptions and experiences with stress, coping, and health promotion programs among African American adolescents with overweight and obesity. Findings from the current study indicate that primary sources of adolescent stress included conflict within the family and among peers, school-related stressors, and in response to various negative emotions (anxiety, depression, anger), which aligns with prior literature [47]. Additional adolescent stressors that are less commonly included in previous studies were described, such as poor home and neighborhood conditions, family health concerns, and pressures related to sports and extracurricular activities. Notably, our findings also demonstrated that nearly half of adolescents reported engaging in avoidant and passive cognitive coping strategies that may exacerbate their experience with stress. Alternatively, only a few adolescents utilized proactive coping strategies, such as cognitive reframing, which has been supported in prior literature as an effective coping strategy [48,49,50]. Moreover, our study extends prior stress and coping studies with the inclusion of salient sources of community support and recommendations for integrating stress management into health promotion programs for African Americans. These findings have important implications on the development of the LEADS health promotion program, which will aim to improve health behaviors and stress management among African American adolescents and their families. 

Adolescents’ primary reports of stressors were tied to interpersonal conflict with family and peers, as well as related to negative emotions. Notably, these stressors are consistent with the Relapse Prevention Model (RPM) and have been shown to challenge one’s ability to effectively cope with stress [51]. Moreover, according to this model of stress and coping, difficulties coping with such stressors can hinder one’s self-efficacy when faced with the stressor again [51]. With limited coping strategies and low self-efficacy to manage stress, there is greater risk for failing to sustain health behavior changes, such as healthy eating or decreased sedentary behavior. These concerns may be particularly salient within our sample of underserved African American adolescents that may have fewer resources to effectively cope with stress [17]. 

Several adolescents in our sample endorsed engaging in poor health behaviors in response to stress. Half reported increased sedentary behavior while others commented on problematic eating behaviors (overeating/undereating, poor food choices, emotional eating, fast food intake) and decreased physical activity. These findings align with prior literature suggesting the relationship between stress and unhealthy behaviors [11,16]. Only a select few adolescents in our sample reported that they engaged in greater levels of physical activity in response to stress. Of note, some adolescents described poor health behaviors their family members engage in while experiencing stress, suggesting that these behaviors may be modeled in the home environment. 

In addition to highlighting relevant stressors and related health behaviors, adolescents reported on prominent coping strategies they utilize. While several adolescents reported approach coping strategies, such as engaging in physical activity, seeking out social support, and utilizing other calming strategies (e.g., listening to music, meditation/mindfulness/prayer), most adolescents engaged in more passive cognitive coping strategies. This included attempts to avoid thinking about or feeling negative emotions related to the stressor, as well as trying to distract oneself from the stressor. Importantly, past research has shown that these types of coping strategies are often linked to more negative outcomes [48]. According to the RPM, cognitive reframing or restructuring is a key strategy that aims to adjust one’s perception of and attitude toward various life stressors and coping behaviors to encourage more proactive (rather than passive) responses to stress [52]. Considering our findings of avoidant coping and passive cognitive coping strategies, it is particularly important for future research to focus on building adaptive stress and coping strategies, such as cognitive reframing, among African American adolescents with overweight and obesity. 

Notably, more than half of adolescents reported the importance of receiving parental support when faced with stress. Specifically, adolescents described the benefit of a supportive home environment and family interactions, by means of emotional support and positive communication, while managing stressors. Building these positive family interactions may be especially important within this sample considering the number of adolescents that reported interpersonal family conflict as a primary stressor. In line with the RPM, maintaining life balance, which includes balancing stressful life experiences with enjoyable, fulfilling activities and interactions, is a critical element to promote healthy outcomes [52,53]. Thus, it will be important for future studies to promote the facilitation of family life balance, as well as incorporate strategies to build family resilience. 

In addition to the value of family support, adolescents also endorsed community and health promotion program supports to help address stress. Specifically, adolescents highlighted the need for financial and tangible support in their community to increase access to resources, as well as repair of community infrastructure (e.g., schools, roads, parks) as a means to reduce stress. The utility of building community centers as a central location for the community to interact and support each other was also emphasized. Notably, financial and tangible support have been associated with improvements in health behaviors, including increased physical activity [54,55]. Recommendations for future programs indicated a desire for integrating health promotion and stress management techniques, with adolescents suggesting programs focused on healthy eating and physical activity, as well as building coping and family skills and receiving support from mental health care providers. Thus, integrating broader contextual factors at the neighborhood and community level may be important for future research focused on health promotion and stress management.

While these findings expand on the limited studies that have explored stress, coping, and health promotion among African American adolescents, these findings may be limited given our small sample size and geographic limitations to the southeast US. Although we purposefully selected our study sample from families that previously completed our research team’s FIT trial, given that they had intervention program experience, our findings may not be generalizable to families that have not participated in a health promotion program. Our research team sought to receive feedback from FIT families that could provide meaningful insight on how to expand future health promotion programs that target treatment-seeking families of similar backgrounds, namely the LEADS trial, informed by their experiences in the FIT trial. Comparisons of the current study demographics with the larger FIT sample indicate that family characteristics are relatively equivalent, apart from a significant difference in the prevalence of parental marital status; thus, indicating that our findings with this subsample may be generalizable to LEADS families in the broader South Carolina community. Importantly, this is one of the first studies to examine stress and coping and recommendations for future trials among high-risk African American adolescents and their families following completion of a health promotion program, and thus makes a novel contribution to current literature. 

Of note, adolescents did not discuss racial discrimination or COVID-19 as stressors in this study, however, the qualitative interviews were conducted between November 2018 and February 2020, and thus before several high-profile cases of racial injustices and the onset of the COVID-19 pandemic in the United States occurred and were highlighted in the media. While racial discrimination has been well-established as a prominent stressor within minority populations [13], it is plausible that families are hesitant to disclose or discuss these stressors in depth in individual interviews. Prior qualitative studies that explicitly questioned African American families’ experiences with race-related stressors found that these stressors did not emerge as a primary theme, and thus prompting these discussions may not necessarily lead to greater disclosure of race-related stressors [42]. The present study had interviewer-participant racial/ethnic discordance, which may have limited our findings by impacting participants’ comfortability or affect during the interview, particularly with disclosure of race-related stressors unfamiliar to the interviewer [39,40]. It will be important for future research to examine concordance among interviewers and participants and how this may impact participant disclosure. Despite these limitations, this study provides unique insights into stress and coping among a high-risk population that will inform future trials.

The current study findings provide novel insights and recommendations to guide the development of the LEADS health promotion trial, which will be conducted in the southeast with African American adolescents with overweight and obesity and their families. Notably, some adolescents highlighted stressors related to poor home and neighborhood environment conditions and community limits in access to healthy foods and resources, which aligns with prior research examining chronic stressors among African Americans [13]. Prior studies also emphasize racial discrimination as a prominent chronic stressor among African American families [13]. Thus, the LEADS trial will aim to provide tailored stress management strategies with a resilience and assets framework to cope with these health inequities that may be particularly salient to underserved African American adolescents and their families. Moreover, our findings point to the elevated endorsement of passive, coping strategies among adolescents, and minimal use of cognitive reframing or countering negative thoughts. Given the abundant research suggesting the benefits of cognitive reframing and restructuring, these stress management techniques will be emphasized in the LEADS trial [52,56]. Furthermore, behavioral skills training, including goal-setting, self-monitoring, and problem-solving, has been shown to play a central role in helping individuals to develop self-efficacy and learn how to overcome obstacles [57,58]. Complementing the use of cognitive restructuring, behavioral skills training will also be implemented in the LEADS trial to help adolescents learn how to anticipate challenges and develop action plans for overcoming stress-related barriers. Taken together, the current study findings have important implications for the LEADS trial and related future studies.

## 5. Conclusions

This qualitative study is one of the first to assess perceptions of how stress relates to healthy eating and physical activity in African American adolescents with overweight and obesity. Adolescents highlighted an interest in building their stress coping skills and their family’s ability to discuss and manage stress, as well as addressing negative psychological and cognitive processes of stress as part of their health needs. Thus, it will be important for future studies, including randomized controlled trials, to assess the integration of both stress management strategies and health promotion approaches in order to promote more active engagement in intervention components. In particular, it is critical that future research incorporates components from the RPM (coping behavior strategies, cognitive reframing, and family life balance) with health promotion efforts that are tailored for high-risk African American adolescent populations.

## Figures and Tables

**Table 1:** Interview Questions.

Core Questions
1. When I say the word “stress,” what does it mean to you?
2. What are the main sources of stress in your life?
3. How does your body feel physically when you are stressed? How do you feel emotionally? How does that affect your health behaviors and your overall health? What about the health of your family?
4. How do you usually cope with feeling stressed?
5. Who do you rely on for support when you are stressed? How do they help you specifically when you are stressed?
6. How does your community help you and your family deal with stress?
7. If you had all the money in the world, how would you change your community to support you and your family in dealing with stress?
8. If you could design a program to support your family maintaining a healthy weight, what would be important things to include in the program for dealing with stress?

**Table 2:** Descriptive data for the subsample ^1^ & the full FIT sample ^2^.

Variable	(*N* = 30) ^1^	(*N* = 241) ^2^	*p*-Value
Adolescent age (years) *M*(*SD*)	15.30 (2.18)	12.83 (1.75)	0.80
Adolescent BMI % *M(SD)*	96.7 (3.90)	96.61 (4.25)	0.89
Adolescent sex (*n*, % female)	15 (50%)	153 (63.50%)	0.15
Parent age (years) *M*(*SD*)	49.72 (10.88)	43.18 (8.65)	0.10
Parent BMI (cm/kg^2^) *M*(*SD*)	37.63 (8.21)	37.48 (8.35)	0.89
Parent sex (*n*, % female)	1 (96.7%)	231 (95.90%)	0.83
Parent Married (*n*, %)	5 (16.7%)	83 (34.4%)	0.05
Parent education (*n*, %)			0.62
9 to 11 years	0 (0%)	6 (2.5%)	
12 years	2 (6.7%)	32 (13.3%)	
Some college	16 (53.3%)	99 (41.1%)	
4-year college	4 (13.3%)	45 (18.7%)	
Professional degree	8 (26.7%)	55 (22.8%)	
Parent Annual Household Income (*n*, %)			0.93
<$10K	2 (6.7%)	36 (14.9%)	
$10–24K	4 (13.3%)	49 (20.3%)	
$25–39K	12 (40%)	64 (26.6%)	
$40–54K	7 (23.3%)	31 (12.9%)	
$55K+	5 (16.6%)	57 (23.7%)	

Note. This table presents comparison demographic information for the subsample used for the current study ^1^ (*N* = 30) and the full FIT trial sample ^2^ (*N* = 241). T-tests and chi-square tests analyses were conducted to determine if there were significant differences in descriptive data between the subsample and the FIT sample.

**Table 3:** Summary of Themes.

Themes (Child, *N* = 30)		Sources	Frequency
Perceptions of Stress	Worry/anxiety	13	15
	Negative emotions	9	14
	Unfavorable circumstances	6	6
	Tension building up	5	7
	Barriers that hold you back	4	6
	Feeling helpless	4	5
	Too many things going on	3	3
	Unexpected difficulties	3	3
Stressors	**School**	**26**	**85**
	**Peer/romantic relationships**	**19**	**55**
	**Interpersonal/family conflict**	**16**	**64**
	Pressure of sports/extracurricular activities	9	38
	Family health concerns	8	14
	Living conditions/neighborhood status	8	12
	Work	7	11
	Worrying about family	6	14
	Lack of time	6	7
	Financial struggles	5	9
	Cognitive overwhelm	5	6
	Lack of social support	2	3
	Personal health concerns	2	2
	Emotional struggles	2	2
Consequences of Stress	**Emotional consequences of stress**	**27**	**76**
	**Physical consequences of stress (i.e., headache)**	**17**	**35**
Health Behaviors	**Increased sedentary behavior**	**15**	**24**
	Loss of appetite	13	22
	Overeating	12	19
	Poor food choices	11	17
	Excessive sleep	8	14
	Decreased physical activity	5	7
	Increased physical activity	4	6
	Emotional eating	4	5
	Lack of sleep	4	7
	Increased fast food consumption	2	3
	Poor parent health behavior (modeling)	2	3
Additional Behaviors	Emotional/cognitive distancing	10	15
	Physical Isolation	9	15
	Increased media use (T.V., Internet, Gaming)	5	5
Coping Strategies			
*Behavioral*	**Physical Activity**	**15**	**27**
	Music	10	22
	Rest/Relaxation	8	9
	Physical Isolation	7	11
	Video/Computer Games	7	7
	Eating habits	6	8
	TV/Movies	6	6
	Reading/writing	5	5
	Phone use (i.e., apps, social media)	5	6
*Cognitive*	Passive Coping (Emotional/Cognitive Distancing + Distraction)	14	24
	Meditation/Mindfulness/Prayer	7	11
	Reflection/contemplation	5	7
	Positive thinking	4	5
	Thought alteration	1	1
	Therapy	1	3
*Social*	**Talking to spouse/partner/friends**	**15**	**19**
	Spending time with friends	11	16
	Spending time with family	4	4
	Volunteering	2	2
Social Support	**Parents**	**21**	**26**
	**Friends**	**20**	**32**
	Lack of community/neighborhood support	13	19
	Extended family	11	13
	Lack of social support	10	17
	School	9	14
	Church group/religious leader (positive)	8	13
	Siblings	6	7
	Neighbors	5	6
	Immediate family	4	5
	Community group	4	4
	Teacher/Professor	4	6
	Romantic partner	2	2
	Sport or recreational team	2	2
	Community government	2	3
Type of Support	**Emotional support**	**20**	**43**
	**Advice (Informational Support)**	**19**	**41**
	**Listening/Talking**	**19**	**35**
	Tangible support	5	8
	Spiritual guidance	4	6
	Financial support	2	2
Community Insights	**Financial support**	**18**	**28**
	Tangible support	14	22
	Building a community center	11	15
	Neighborhood collective efficacy	7	10
	Group environment	6	9
	Mental health services	5	8
	Peer support	3	4
Program Recommendations	**Healthy eating**	**16**	**24**
	Coping skills	9	12
	Physical activity	8	9
	Therapist/counselor	7	10
	Family skills	4	10

Note. Themes endorsed by half of participants or more (*N* = 15+) were noted with bolded text.

## Data Availability

The data presented in this study are available upon request from the corresponding author.
